# Supplementary material for: Expression Level of Mature miR172 in Wild Type and StSUT4-Silenced Plants of Solanum tuberosum Is Sucrose-Dependent
Source: Int J Mol Sci. 2021 Feb 1;22(3):1455. doi: 10.3390/ijms22031455 (PMC7867175; doi:10.3390/ijms22031455)
Supplement: Supplementary file 1 [file ijms-22-01455-s001.pdf]

Supplementary Figure S1

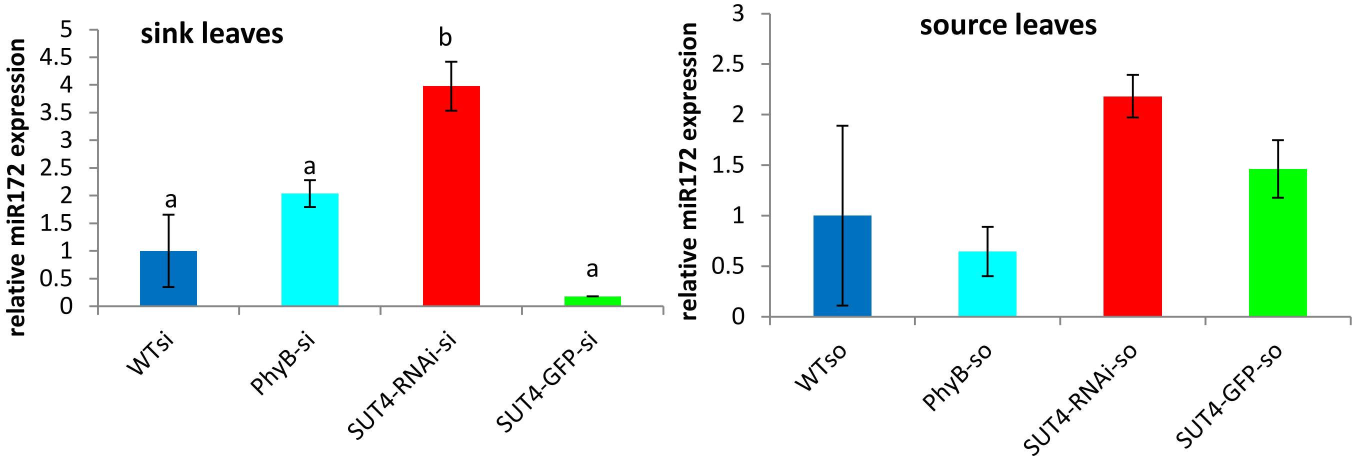

Supplementary Figure S1. Real time qPCR of miR172 in sink (left) and source leaves (right) of *StSUT4*-RNAi plants (*Solanum tuberosum* ssp. tuberosum variety Désirée) revealed significantly increased levels of miR172 *in StSUT4* silenced Désirée plants as well. PhyB antisense potato plants were kindly provided by Salomé Prat. 5S rRNA served as reference gene for relative quantification. SEM is given.

Supplementary Figure S2

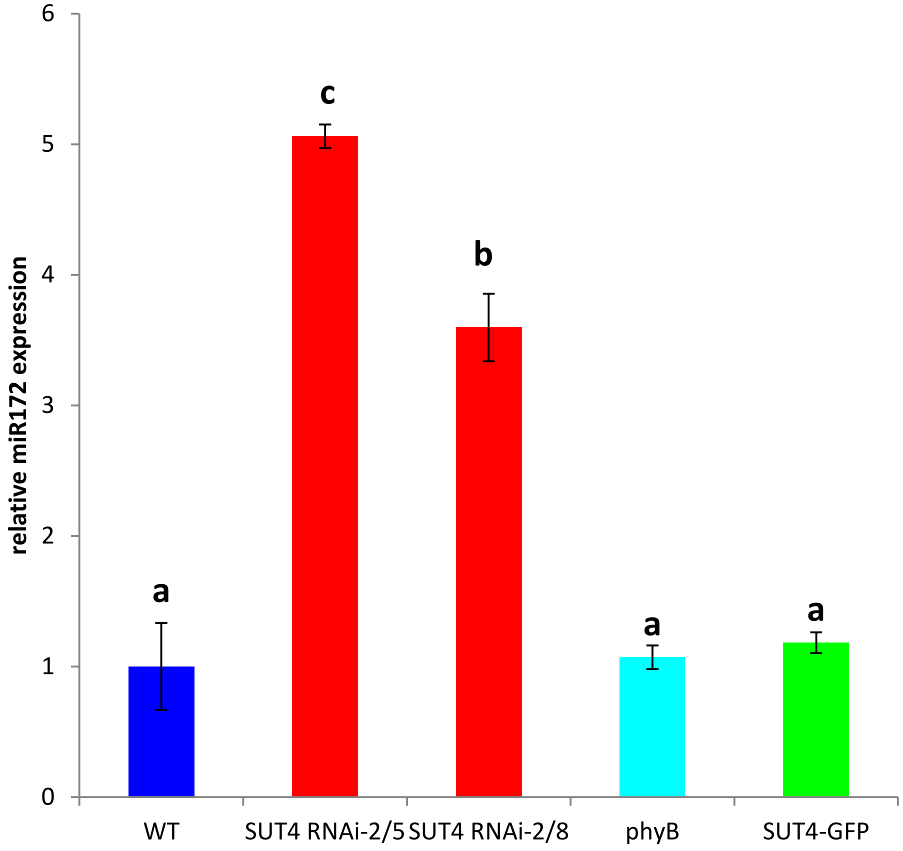

Supplementary Figure S2. Real time qPCR of miR172 in the shoot apical meristem of 10 weeks old plants revealed significantly increased levels of miR172 in *StSUT4*-silenced plants. 5S rRNA served as reference gene for relative quantification. *StSUT4*-inhibition was performed in *Solanum tuberosum* ssp. andigena and the level of miR172 compared to andigena WT plants. The SEM is given.

Supplementary Figure S3

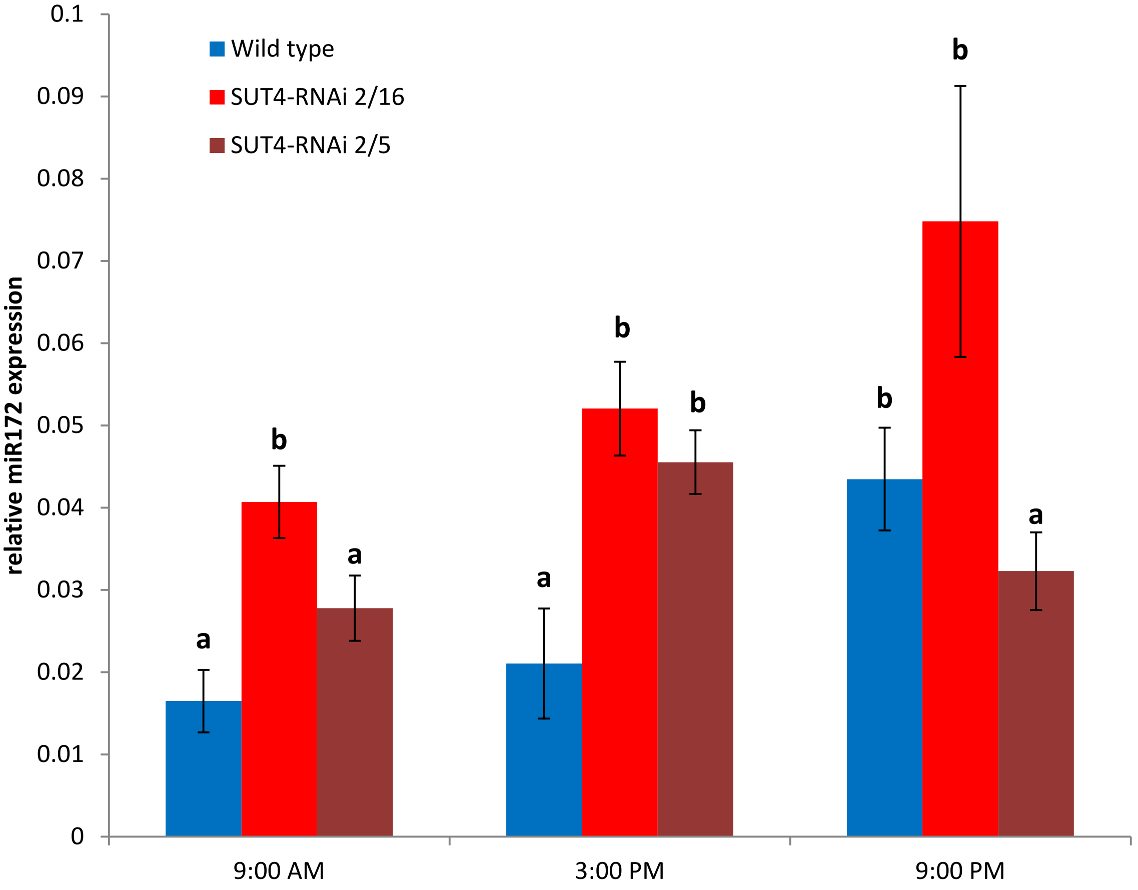

Supplementary Figure S3. Quantification of miR172 in source leaves of andigena wild type as well as StSUT4-RNAi plants at different time points of the day show a diurnal expression pattern. Plants were grown under long day conditions in the green house with light form 6 am to 10 pm. Note that the level of miR172 increases with increasing amounts of solubel sugars in source leaves as measures previously (Chincinska et al. 2008). Two biological and two technical replicates were averaged for each sample. Quantification was performed using 5SrRNA as a reference. SEM is given.

Supplementary Figure S4

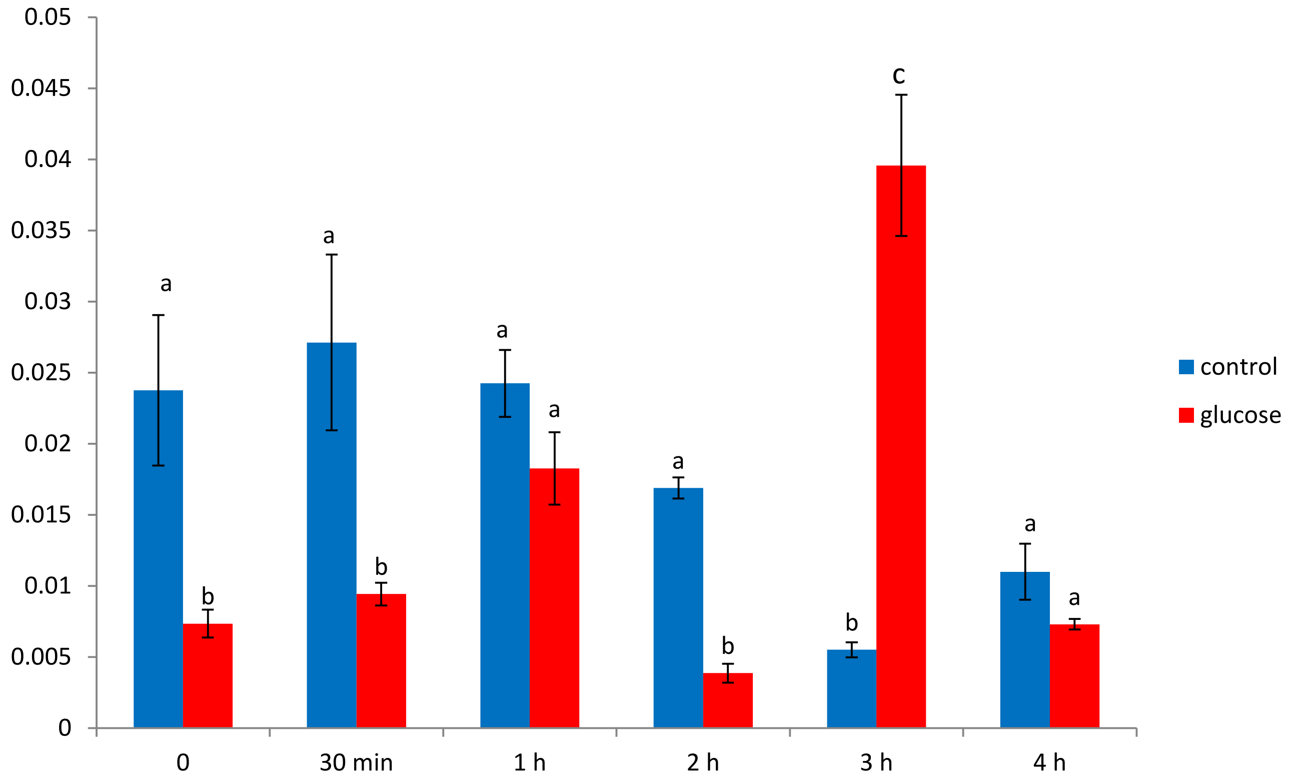

Supplementary Figure S4. Quantification of miR172 in mature leaves in response to glucose feeding. Short time petiole experiment in the presence or absence of glucose was conducted with source leaves of 6 weeks old potato wild type plants (*Solanum tuberosum* variety Désirée). Glucose was supplied at a concentration of 100 mM in 2.5 mM EDTA. Note that miR172 levels in the absence of sugars might oscillate diurnally during the day. Quantification was performed using 5SrRNA as a reference. SEM is given.
